# Palbociclib treatment alters nucleotide biosynthesis and glutamine dependency in A549 cells

**DOI:** 10.1186/s12935-020-01357-x

**Published:** 2020-07-01

**Authors:** Lindsey R. Conroy, Pawel Lorkiewicz, Liqing He, Xinmin Yin, Xiang Zhang, Shesh N. Rai, Brian F. Clem

**Affiliations:** 1 Department of Biochemistry and Molecular Genetics, University of Louisville, Louisville, KY USA; 2 Diabetes and Obesity Center, Christina Lee Brown Envirome Institute, Louisville, KY USA; 3 Department of Chemistry, Center for Regulatory and Environmental Analytical Metabolomics, University of Louisville, Louisville, KY USA; 4 Department of Bioinformatics and Biostatistics, University of Louisville, Louisville, KY USA; 5 Biostatistics and Bioinformatics Facility, James Graham Brown Cancer Center, University of Louisville, Louisville, KY USA; 6 James Graham Brown Cancer Center, Louisville, KY USA; 7 Present Address: Department of Neuroscience, University of Kentucky, Lexington, KY USA

**Keywords:** RB, Palbociclib, Metabolism, Glutaminolysis, PPP, Lung cancer

## Abstract

**Background:**

Aberrant activity of cell cycle proteins is one of the key somatic events in non-small cell lung cancer (NSCLC) pathogenesis. In most NSCLC cases, the retinoblastoma protein tumor suppressor (RB) becomes inactivated via constitutive phosphorylation by cyclin dependent kinase (CDK) 4/6, leading to uncontrolled cell proliferation. Palbociclib, a small molecule inhibitor of CDK4/6, has shown anti-tumor activity in vitro and in vivo, with recent studies demonstrating a functional role for palbociclib in reprogramming cellular metabolism. While palbociclib has shown efficacy in preclinical models of NSCLC, the metabolic consequences of CDK4/6 inhibition in this context are largely unknown.

**Methods:**

In our study, we used a combination of stable isotope resolved metabolomics using [U-^13^C]-glucose and multiple in vitro metabolic assays, to interrogate the metabolic perturbations induced by palbociclib in A549 lung adenocarcinoma cells. Specifically, we assessed changes in glycolytic activity, the pentose phosphate pathway (PPP), and glutamine utilization. We performed these studies following palbociclib treatment with simultaneous silencing of *RB1* to define the pRB-dependent changes in metabolism.

**Results:**

Our studies revealed palbociclib does not affect glycolytic activity in A549 cells but decreases glucose metabolism through the PPP. This is in part via reducing activity of glucose 6-phosphate dehydrogenase, the rate limiting enzyme in the PPP. Additionally, palbociclib enhances glutaminolysis to maintain mitochondrial respiration and sensitizes A549 cells to the glutaminase inhibitor, CB-839. Notably, the effects of palbociclib on both the PPP and glutamine utilization occur in an RB-dependent manner.

**Conclusions:**

Together, our data define the metabolic impact of palbociclib treatment in A549 cells and may support the targeting CDK4/6 inhibition in combination with glutaminase inhibitors in NSCLC patients with RB-proficient tumors.

## Background

Lung cancer is among the most frequently diagnosed cancers and is the leading cause of cancer-related death worldwide, with an estimated 1.6 million deaths each year [1–3]. Of its subtypes, non-small cell lung cancer (NSCLC) accounts for 85% of all lung cancer diagnoses [2]. One of the hallmark events in NSCLC pathogenesis is deregulation of the cell cycle [4, 5]. Transition from G1 to S phase during cell cycle progression is tightly regulated by phosphorylation of the retinoblastoma protein tumor suppressor (RB) by the cyclin dependent kinase (CDK) 4/6-Cyclin D complex. Activity of the CDK4/6-Cyclin D complex is strongly inhibited by p16 which induces cell cycle arrest. In the majority of NSCLC cases, p16 is mutated, deleted, or epigenetically silenced, resulting in constitutive phosphorylation of RB and uncontrolled cell cycle progression [4, 5]. Pharmacologically, CDK4/6 inhibition phenocopies p16 activity [6, 7].

Highly selective and potent small-molecule CDK4/6 inhibitors have shown anti-tumor activity both in vitro and in vivo [6, 7]. Among these inhibitors, palbociclib (PD-0332991) has been FDA-approved for the treatment of women with breast cancer [8]. Recently, palbociclib has shown efficacy either alone or as combination therapy in preclinical studies of NSCLC [9–13]. Mechanistically, the functional consequences of CDK4/6 inhibition apart from cell cycle arrest in NSCLC are largely unknown; yet, metabolic reprogramming in response to palbociclib has revealed unique targetable vulnerabilities in several cancers including pancreatic, colorectal, and leukemia [14–16]. As such, we aimed to characterize the metabolic phenotype induced by palbociclib in NSCLC.

Herein, we report that palbociclib treatment decreases nucleotide biosynthesis and increases glutamine utilization without altering glycolysis in A549 lung adenocarcinoma cells. Specifically, palbociclib-induced glutamine dependency sensitizes A549 cells to glutaminase inhibition. Together, these data expand our knowledge of understanding of the metabolic consequences of CDK4/6 inhibition in NSCLC.

## Methods

### Cell culture

The A549 lung adenocarcinoma cells were obtained from ATCC and cultured in DMEM (Gibco, Cat. No. 11965126) supplemented with 10% fetal bovine serum (Gibco, Cat. No. 10438034) and 50μg/mL gentamicin sulfate (Gibco, Cat. No. 15750-078). Cells were maintained at 5% CO_2_ at 37 °C. Palbociclib (PD-0332991) (Cat. No. S1116) and CB-839 (Cat. No. S7655) were purchased from Selleck Chemicals.

### Cell transfections

Using Lipofectamine RNAiMAX reagent (Invitrogen, Cat. No. 13778150), A549 cells were transiently transfected with control siRNA (Ambion, Silencer Select Negative Control #2, Cat. No. 4390826) or siRNA targeted to RB1 (Ambion, Silencer Select s523, Cat. No. 4390824) for 24 h prior to palbociclib treatment.

### Cell proliferation and viability

A549 cells were seeded at 120,000 in 6-well plates (Corning, Cat. No. 3516), followed by transfection and treatment with 1 μM palbociclib or vehicle control. For glutaminase inhibition studies, cells were seeded at 25,000 in 24-well plates (Corning, Cat. No. 3526), followed by 25 nM CB-839 treatment. Forty-eight hours post-treatment, cell number was determined by Trypan Blue (Gibco, Cat. No. 15250-061) exclusion and enumeration using a hemocytometer.

### Glucose uptake

A549 cells were seeded at 120,000 in 6-well plates (Corning, Cat. No. 3516). Cells were transfected and treated with 1 μM palbociclib or vehicle control for 48 h prior to performing the assay. Cells were then incubated in glucose-free DMEM (Gibco, Cat. No. 11966-025) for 30 min, followed by incubation with 5 μL of 2-[^14^C]-deoxyglucose (0.25 μCi/mL; Perkin Elmer, Cat. No. NEC720A050UC) and three subsequent washes with ice-cold glucose-free DMEM. Lysates were collected in 500 μL of 0.1% SDS and scintillation counts (counts/min) were measured using 350 μL of cell lysate on a Tri-Carb 2910 liquid scintillation analyzer (Perkin Elmer Life Sciences). Protein concentration was determined using the BCA assay (Pierce, Cat. No. 23225) according to the manufacturer’s protocol. Counts were normalized to μg of protein.

### Glycolysis stress test

A549 cells were seeded at 120,000 in 6-well plates (Corning, Cat. No. 3516). Cells were transfected and treated with 1 μM palbociclib or vehicle control for 48 or 120 h prior to performing the assay. ECAR measurements were conducted using a Seahorse XFe96 analyzer according to manufacturer’s protocol. One day prior to performing the assay, cells were reseeded at 35,000 in XFe96 cell culture plates and incubated 5% CO_2_ at 37 °C. One hour prior to analysis, growth medium was replaced with assay medium (DMEM minus phenol red and sodium bicarbonate (Corning, Cat. No. 90-013-PB) that is supplemented with 2 mM l-glutamine, pH 7.4) and incubated in a non-CO_2_ incubator. During the assay, 10 mM glucose (Sigma, Cat. No. G8270), 1 μM oligomycin (Sigma, Cat. No. 495455), and 50 mM 2-deoxyglucose (Sigma, Cat. No. D8375) were sequentially injected into each well in accordance with standard protocols. Absolute rates (mpH/min) were normalized to μg of protein.

### Mito stress test

A549 cells were seeded at 120,000 in 6-well plates (Corning, Cat. No. 3516). Cells were transfected and treated with 1 μM palbociclib or vehicle control for 48 h prior to performing the assay. OCR measurements were conducted using a Seahorse XFe96 analyzer according to manufacturer’s protocol. One day prior to performing the assay, cells were reseeded at 35,000 in XFe96 cell culture plates and incubated in 5% CO_2_ at 37 °C. One hour prior to analysis, growth medium was replaced with assay medium (DMEM minus phenol red and sodium bicarbonate (Corning, Cat. No. 90-013-PB) that is supplemented with 1 mM pyruvate, 2 mM l-glutamine, and 10 mM glucose, pH 7.4) and incubated in a non-CO_2_ incubator. During assay, 1 μM oligomycin (Sigma, Cat. No. 495455), 2 μM FCCP (Sigma, Cat. No. C2920), and 1 μM rotenone/antimycin A (Sigma Cat. No. R8875 and A8674) were sequentially injected into each well in accordance with standard protocols. Absolute rates (p moles/min) were normalized to μg of protein.

### Mito fuel flexibility assay

A549 cells were seeded at 120,000 in 6-well plates (Corning, Cat. No. 3516). Cells were transfected and treated with 1 μM palbociclib or vehicle control for 48 h prior to performing the assay. OCR measurements were conducted using a Seahorse XFe96 analyzer according to manufacturer’s protocol. One day prior to performing the assay, cells were reseeded at 35,000 in XFe96 cell culture plates and incubated 5% CO_2_ at 37 °C. One hour prior to analysis, growth medium was replaced with assay medium (DMEM minus phenol red and sodium bicarbonate (Corning, Cat. No. 90-013-PB) that is supplemented with 1 mM pyruvate, 2 mM l-glutamine, and 10 mM glucose, pH 7.4) and incubated in a non-CO_2_ incubator. Capacity and dependency for fatty acid, glutamine, and glucose oxidation were determined through injections of the following inhibitors in accordance with standard protocols: 4 μM etomoxir (Sigma, Cat. No. E1905) (inhibits long chain fatty acid oxidation), 3 μM BPTES (Sigma, Cat. No. SML0601) (inhibits glutamine oxidation), and 2 μM UK5099 (Sigma, Cat. No. PZ0160) (inhibits glucose oxidation). The percent capacity for each fuel source was determined by dividing the observed OCR under inhibition of the two other pathways by the OCR observed when all pathways are inhibited. The percent dependency for each fuel source was determined by dividing the OCR observed when that specific pathway is inhibited by the OCR observed when all pathways are inhibited. Absolute rates (p moles/min) were normalized to μg of protein.

### Glucose 6-phosphate dehydrogenase activity assay

A549 cells were seeded at 120,000 in 6-well plates (Corning, Cat. No. 3516). Cells were transfected and treated with 1 μM palbociclib or vehicle control for 48 h prior to performing the assay. The activity of G6PD from cell lysates was measured following the manufacturer’s protocol using the Glucose-6-Phosphate Dehydrogenase Activity Assay Kit (Sigma, Cat. No. MAK015-1KT) and normalized to μg of protein.

### [U-^13^C]-glucose tracer studies

Cells were seeded at 1 × 10^6^ cells in 10 cm dishes (Corning, Cat. No. 430167) and treated with palbociclib or vehicle control for 48 h. Cells were then labeled for 6 h with DMEM supplemented with 1 g/L [U-^13^C]-glucose (Sigma, Cat. No. 38974) and 10% dialyzed fetal bovine serum (Gibco, Cat. No. A3382001). Cells were washed three times in ice-cold 1× PBS and quenched with acetonitrile. Metabolites were extracted in acetonitrile:water:chloroform (2 mL:1 mL:740 µL). Samples were centrifuged at 3000×*g* for 20 min at 4 °C to separate the polar, lipid, and cell debris layers. The remaining cell debris was re-extracted with 500 µL chloroform:methanol:butylated hydroxytoluene (2:1:1 mM) and centrifuged at 22,000×*g* for 20 min at 4 °C. The residual polar and lipid fractions were combined with their respective fractions from the first extraction. The polar fraction was vacuum-dried by lyophilization. The dried sample was dissolved in 100 µL 50% acetonitrile and vigorously vortex-mixed for 3 min. After centrifugation at 14,000 rpm and 4 °C for 20 min, 80 µL of supernatant was collected for 2DLC-MS/MS analysis.

## 2DLC-MS/MS analysis and data processing

All samples were analyzed in a random order on a Thermo Q Exactive HF Hybrid Quadrupole-Orbitrap Mass Spectrometer coupled with a Thermo DIONEX UltiMate 3000 HPLC system (Thermo Fisher Scientific, Waltham, MA, USA). The UltiMate 3000 HPLC system was equipped with a reverse phase chromatography (RPC) column and a hydrophilic interaction chromatography (HILIC) column that were configured in parallel to form a parallel 2DLC-MS system [17]. To obtain full MS data, every sample was analyzed by the parallel 2DLC-MS in positive mode (+) and negative mode (−), respectively. For metabolite identification, one unlabeled sample in each sample group was analyzed by 2DLC-MS/MS in positive mode (+) and negative mode (−) to acquire MS/MS spectra at three collision energies (20, 40 and 60 eV).

### Data analysis for 2DLC-MS/MS

Full MS.raw files were first converted to.mzML format with msConvert tool, a part of an open-source ProteoWizard suite, described in detail by Adusumilli and Mallick [18]. Isotopologue peak deconvolution and assignments were performed using El-MAVEN (Elucidata) [19]. Peaks were assigned using a metabolite list generated and verified using full scan MS and MS/MS spectra of unlabeled samples, as described previously [20–22]. The metabolite list contained metabolite names and corresponding molecular formulae used to generate theoretical m/z values for all possible isotopologues, and retention times for each metabolite. El-MAVEN parameters for compound library matching were as follows: EIC Extraction Window ± 7 ppm; Match Retention Time ± 0.60 min. For ^13^C isotopologue peak detection, the software criteria were set as follows: Minimum Isotope-parent correlation 0.20; Isotope is within 5 scans of parent; Abundance threshold 1.0; Maximum Error To Natural Abundance 100%. All assignments were visually inspected and compared to unlabeled samples for reference. The peak list with corresponding abundances was exported to a comma-separated (CSV) file and uploaded to the Polly workflow (Elucidata) for natural abundance correction and calculation of total pool size for each metabolite (by summing peak areas of each detected isotopologue) using Polly Isocorrect module. Finally, the data were downloaded and plotted using Microsoft Excel and GraphPad Prism software.

### Real time-PCR

Total RNA was isolated from cell pellets using the RNeasy Mini Kit (Qiagen, Cat. No. 74104) according to the manufacturer’s protocol. The resulting total RNA (1μg) was converted to cDNA using the High-Capacity RNA-to cDNA kit (Applied Biosystems, Cat. No. 4387406). Gene expression was determined by qPCR using the following Taqman Gene Expression Assays: *RB1* (Hs01078066_m1), *RRM1* (Hs01040698_m1), *RRM2* (Hs00357247_g1), *PRPS2* (Hs00267624_m1), *G6PD* (Hs0166169_m1), *ASCT2* (Hs01056542_m1), *GLS* (Hs01014020_m1), and *GLUD1* (Hs01632647_g1). *β*-*actin* (Hs01060665_g1) was used as an internal control. Data are reported as the log (base 2) of the fold change with respect to siNeg treated with vehicle control.

### Immunoblotting

Whole-cell lysates were prepared in RIPA lysis buffer (Pierce, Cat. No. 89900) containing protease and phosphatase inhibitors. 20 μg of protein was separated on 10% Bolt Bis–Tris gels (Invitrogen, Cat. No. nw00102box) and transferred to PVDF membrane. Membranes were blocked with 5% BSA (Fisher Bioreagents, Cat. No. BP1600-100) in TBS-T and probed with 1:1000 dilution of anti-phospho-Rb (Cell Signaling, #3590S) overnight at 4 °C. Membranes were washed and incubated with HRP-conjugated anti-rabbit (1:5000) secondary antibodies (Invitrogen, Cat. No. 32260) for 1 h. Protein detection was performed by ECL chemiluminescent reagent (GE Healthcare Amersham, Cat. No. RPN2232) exposure. 1:20,000 dilution of anti-β-actin antibody (Sigma, A2228) was used to assess protein loading.

### Statistical analysis

Statistical analyses were carried out using GraphPad Prism. As described in their respective figure legends, analyses were performed using student’s t-test and two-way ANOVA with Sidak’s post hoc multiple comparisons. Within the two-factor ANOVA, interaction term was primarily included in the analysis and was dropped if found not to be significant. Statistical significances between any two groups are as follows: *p < 0.05, **p < 0.01, and ***p < 0.001.

## Results

### Palbociclib treatment does not alter glycolysis in A549 cells

**Fig. 1:**
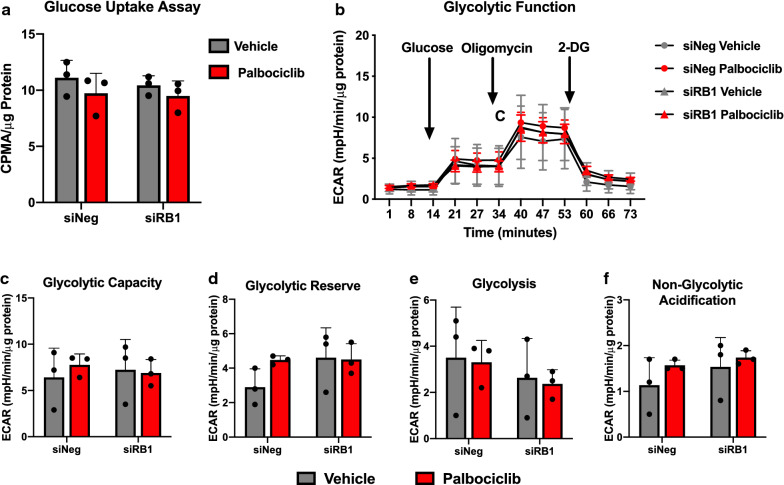
Palbociclib does not alter glucose uptake or glycolytic function in A549 cells. **a** Glucose uptake assessed by intracellular 2-[^14^C]-deoxyglucose in A549 cells following knockdown of RB and 48-h treatment with 1 μM palbociclib. **b** Glycolysis stress test measured in A549 cells following knockdown of RB and 48-h treatment with 1 μM palbociclib using a Seahorse XFe96 Analyzer. **c**–**f** ECAR assessment of **c** glycolytic capacity, **d** glycolytic reserve, **e** glycolysis, and **f** non-glycolytic acidification. Values represent mean ± SD, analyzed by two-way ANOVA with Sidak’s post hoc multiple comparisons (n = 3, independent experiments)

To assess the role of CDK4/6 inhibition on metabolism in NSCLC, we utilized A549 lung adenocarcinoma cells. While these cells express wild-type RB, they harbor a p16^INK4A^ deletion that results in constitutive RB hyperphosphorylation and inactivation. Treatment with palbociclib inhibits the phosphorylation of RB and results in decreased cell proliferation (Additional file 1: Figure S1A, B). Recent studies have demonstrated that palbociclib alters glucose metabolism in multiple cancer subtypes [23–25]. To monitor the effect of palbociclib on glucose uptake, we performed radiolabeled glucose uptake assay using 2-[^14^C]-deoxyglucose in A549 cells. As CDK4/6 inhibition can have metabolic effects independent of RB, these studies were performed following palbociclib treatment with simultaneous knockdown of *RB1* (Additional file 1: Figure S2). We observed no significant change in glucose uptake upon palbociclib treatment in A549 cells (Fig. 1a). To assess glycolytic function in response to palbociclib treatment, we performed a glycolysis stress test using the Seahorse XFe96 analyzer. Similar to glucose uptake, we observed no difference in the extracellular acidification rate (ECAR) upon palbociclib treatment (Fig. 1b–f), suggesting palbociclib does not alter glycolysis in A549 cells.

**Fig. 2:**
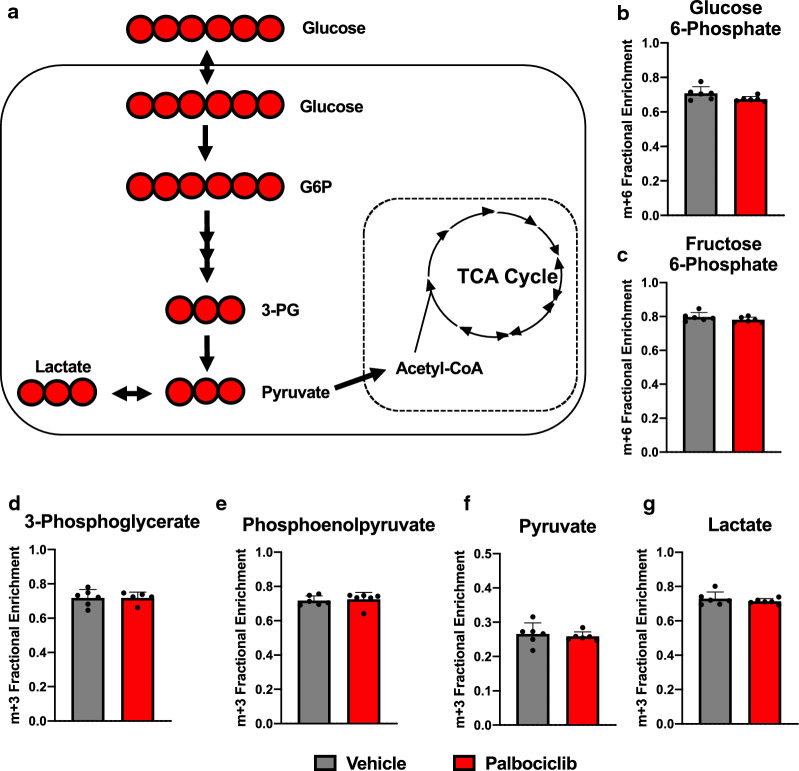
Palbociclib does not alter glucose carbon incorporation into glycolytic intermediates in A549 cells. **a** Cartoon of [U-^13^C]-glucose fate mapping though glycolysis. Red circles indicate ^13^C. Fractional enrichment of m + 6 labeled **b** glucose 6-phosphate and **c** fructose 6-phosphate, and m + 3 labeled **d** 3-phosphoglycerate, **e** phosphoenolpyruvate, **f** pyruvate, and **g** lactate following 48-h treatment with 1 μM palbociclib. Values represent mean ± SD, analyzed by unpaired student’s t-test (n = 6, technical replicates)

To define global changes in glucose utilization resulting from palbociclib treatment, we performed [U-^13^C]-glucose tracer studies. Utilization of ubiquitously labeled glucose results in the intracellular generation of ^13^C labeling of the hexose and triose sugar intermediates within the glycolytic pathway; leading to fully labeled pyruvate (m + 3) and lactate (m + 3) (Fig. 2a) Palbociclib has been shown to increase glycolytic flux in both breast and pancreatic cancers [14, 24, 25]; however, consistent with our glycolysis stress test findings, palbociclib did not alter glucose carbon incorporation into glycolytic intermediates in A549 cells (Fig. 2b–g). Our data suggest that the palbociclib-mediated effects on glycolysis may be tissue-specific.

### CDK4/6 inhibition decreases glucose flux through the PPP

**Fig. 3:**
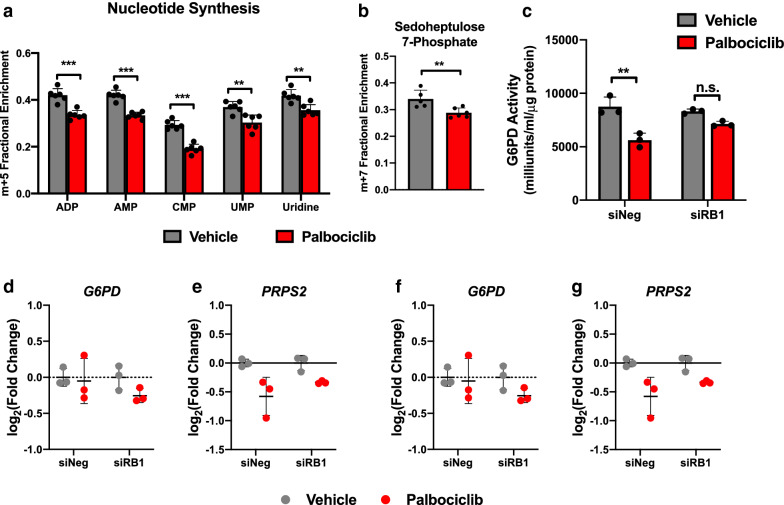
Palbociclib decreases glucose carbon incorporation into PPP intermediates, in part, via decreased G6PD activity. **a** Fractional enrichment of m + 5 labeled nucleotides and nucleosides, and **b** m + 7 sedoheptulose 7-phosphate following 48-h treatment with 1 μM palbociclib. Values represent mean ± SD, analyzed by unpaired student’s t-test (n = 6, technical replicates). Statistical significances between each group are as follows: **p < 0.01 and ***p < 0.001. **c** G6PD activity assessed in A549 cells following knockdown of RB and 48-h treatment with 1 μM palbociclib. **d**–**g** qPCR analysis of glucose 6-phosphate dehydrogenase (*G6PD*), phosphoribosyl pyrophosphate synthetase 2 (*PRPS2*), and ribonucleotide reductase 1/2 (*RRM1/2*) in A549 cells following knockdown of RB and 48-h treatment with 1 μM palbociclib. **c**–**g** Values represent mean ± SD, analyzed by two-way ANOVA with Sidak’s post hoc multiple comparisons (n = 3, independent experiments). Statistical significances between each group are as follows: **p < 0.01

We then examined whether glucose metabolism within tangential pathways, such as the pentose phosphate pathway, may be altered by palbociclib treatment. Metabolism of fully labeled ^13^C-glucose generates different metabolite labeling patterns within PPP intermediates, including the 5-carbon labeled ribose moiety (m + 5) on de novo synthesized nucleotides from ribose-5-phosphate as well as 7 labeled carbon sedoheptulose-7-phosphate (m + 7). We observed decreased m + 5 isotopologue labeling of several nucleotides (Fig. 3a) as well as diminished glucose carbon incorporation into sedoheptulose 7-phosphate (Fig. 3b). Together, these data suggest palbociclib decreases glucose metabolism through the PPP. Consistent with our labeling data, palbociclib decreased glucose 6-phosphate dehydrogenase (G6PD) activity in an RB-dependent manner (Fig. 3c). Additionally, we measured expression of *G6PD* and other genes within nucleotide synthesis including phosphoribosyl pyrophosphate synthetase 2 (*PRPS2*) and ribonucleotide reductase 1/2 (*RRM1/2*). Palbociclib decreased expression of *RRM2* in an RB-dependent manner but does not significantly alter expression of these other genes within nucleotide synthesis (Fig. 3d–g).

### Palbociclib increases oxygen consumption in A549 cells

**Fig. 4:**
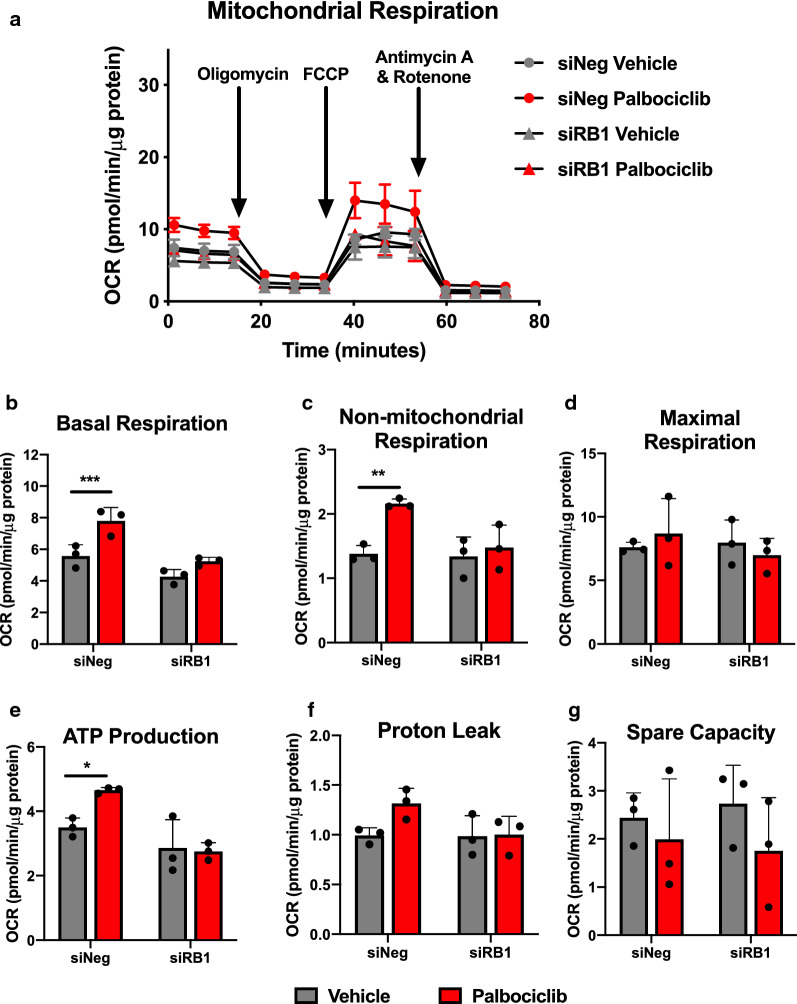
Palbociclib increases mitochondrial respiration in A549 cells. **a** Oxygen consumption rate (OCR) measured in A549 cells following knockdown of RB and 48-h treatment with 1 μM palbociclib. **b**–**g** OCR assessment of **b** basal respiration, **c** non-mitochondrial respiration, **d** maximal respiration, **e** ATP production, **f** proton leak, and **g** spare capacity. Values represent mean ± SD, analyzed by two-way ANOVA (n = 3, independent experiments). Statistical significances between vehicle and palbociclib treatment are as follows: *p = 0.034, **p = 0.01, and ***p = 0.002

CDK4/6 inhibition has been reported to increase both mitochondrial mass and oxygen consumption in pancreatic cancer [14]. As such, we assessed mitochondrial activity by measurement of the oxygen consumption rate (OCR) in A549 cells following palbociclib treatment. Consistent with other cancer types [14, 15], we observed a significant increase in several parameters of mitochondrial activity including basal (p = 0.002) and non-mitochondrial respiration (p = 0.01) and ATP production (p = 0.034) (Fig. 4). While two-way ANOVA revealed no interaction between palbociclib treatment and RB status (p = 0.113), palbociclib increased basal OCR 1.4-fold, which was reduced to 1.2-fold in the absence of RB.

**Fig. 5:**
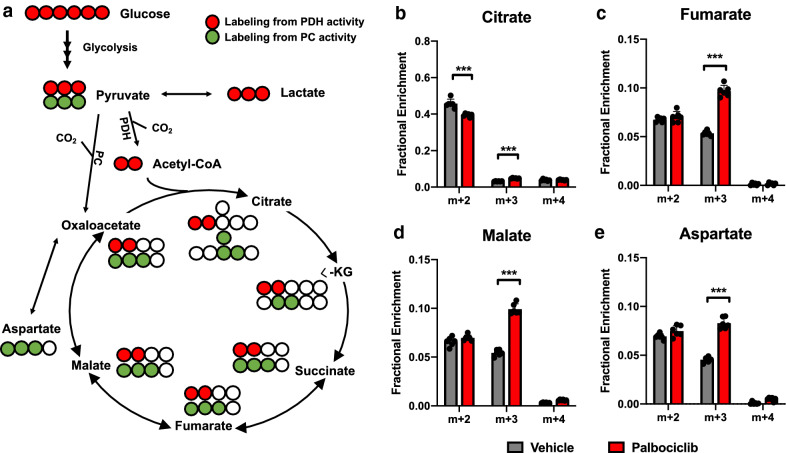
Palbociclib increases PC-mediated anaplerosis in A549 cells. **a** Cartoon of [U-^13^C]-glucose fate mapping through the 1st turn of the TCA cycle. Red circles are ^13^C labeling indicative of pyruvate dehydrogenase activity, green circles are ^13^C labeling indicative of pyruvate carboxylase activity, and white circles are unlabeled ^12^C. **b**–**e** Fractional enrichment of the TCA metabolites **b** citrate, **c** fumarate, **d** malate, and **e** aspartate in A549 cells following 48-h treatment with 1 μM palbociclib. Values represent mean ± SD, analyzed by unpaired student’s t-test for each metabolite isotopologue (vehicle vs. palbociclib) (n = 6, technical replicates). Statistical significances between each group are as follows: ***p < 0.001

To determine if the increase in basal oxygen consumption upon palbociclib treatment is the result of increased glucose oxidation, we measured TCA cycle metabolites following [U-^13^C]-glucose labeling in A549 cells. Pyruvate (m + 3) generated from glycolysis can be metabolized to lactate by lactate dehydrogenase (LDH) or oxidized in the TCA cycle through pyruvate dehydrogenase (PDH) or pyruvate carboxylase (PC). PDH entry of pyruvate carbon is indicated by m + 2 (1st turn)/m + 4 (2nd turn) isotopologues, while PC activity is observed by m + 3 isotopologue labeling of TCA intermediates (Fig. 5a). While there was no consistent effect on PDH-mediated pyruvate carbon entry (m + 2 or m + 4), we found that palbociclib increased m + 3 labeling of several TCA metabolites, suggesting palbociclib may increase PC activity for anaplerosis in A549 cells (Fig. 5b–e).

### Palbociclib sensitizes A549 cells to glutaminase inhibition in an RB-dependent manner

**Fig. 6:**
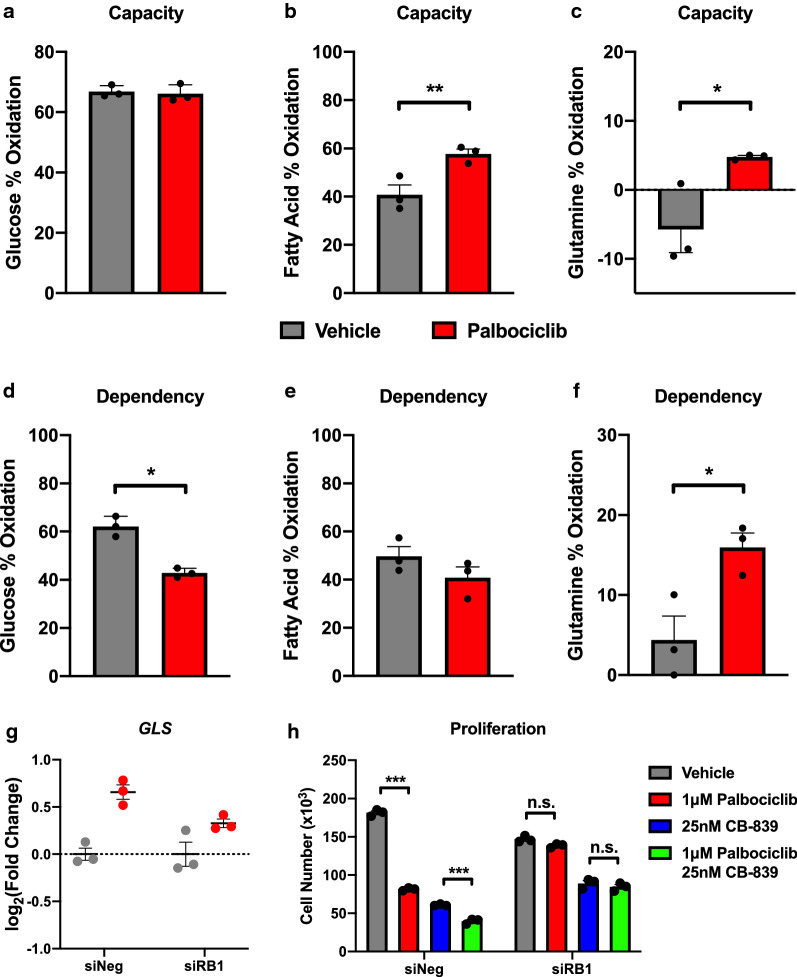
Palbociclib increases glutamine dependency and sensitizes A549 cells to glutaminase inhibition. **a** Glucose, **b** fatty acid, and **c** glutamine capacity, and **d** glucose, **e** fatty acid, and **f** glutamine dependency measured by the Mito Fuel Flex assay in A549 cells following 48-h treatment with 1 μM palbociclib. For (**a**–**f**), values represent mean ± SD, analyzed by unpaired student’s t-test (n = 3, independent experiments). Statistical significances between each group are as follows: *p < 0.05 and **p < 0.01. **g** qPCR analysis of glutaminase (*GLS*) following knockdown of RB and 48-h treatment with 1 μM palbociclib. **h** Cell proliferation following knockdown of RB and 48-h treatment with 1 μM palbociclib and/or 25 nM CB-839. For (**g**, **h**), values represent mean ± SD, analyzed by two-way ANOVA with Sidak’s post hoc multiple comparisons (n = 3, independent experiments). Statistical significances between each group are as follows: ***p < 0.001

We next sought to determine whether palbociclib alters the capacity to metabolize or the dependency on specific TCA fuels such as glucose, glutamine, or fatty acids for mitochondrial function using the Mito Fuel Flex assay on the Seahorse XFe96 analyzer. Palbociclib had no effect on the overall capacity of A549 cells to utilize glucose but did decrease the cells’ dependency on glucose to maintain basal respiration, indicating glucose is not the sole fuel source for the TCA cycle in palbociclib treated cells (Fig. 6a, d). While palbociclib increases the capacity to oxidize fatty acids, it has no effect on the cells’ dependency for fatty acid oxidation, suggesting that inhibition of fatty acid oxidation would have no effect on the viability of palbociclib treated cells (Fig. 6b, e). Notably, we observed an increase in both glutamine capacity and dependency in A549 cells upon palbociclib treatment (Fig. 6c, f).

The enzyme glutaminase converts glutamine to glutamate and is highly expressed in cancer cells [26–28]. CB-839 is a highly potent glutaminase inhibitor that has shown anti-tumor activity in both in vitro and in vivo models of lung cancer [29]. We hypothesized that the observed increase in glutamine dependency may sensitize palbociclib treated cells to glutaminase inhibition. Although palbociclib does not significantly alter glutaminase (*GLS*) gene expression (Fig. 6g), we observed a significant proliferative decrease in cells treated in combination with the glutaminase inhibitor CB-839 and palbociclib compared with palbociclib or CB-839 alone (Fig. 6h). Additionally, statistical analysis found a significant interaction between inhibitor sensitivity and RB status (p < 0.0001), suggesting RB expression is necessary for palbociclib to exert its effect on glutamine dependency. Together, our data highlight that palbociclib-induced metabolic adaptations have the potential to be therapeutically exploited.

## Discussion

While targeting cancer metabolism for therapeutic intervention has been extensively studied [30–34], there is a growing appreciation for cell cycle proteins that display metabolic regulatory functions [6, 35–38]. Additionally, with the increase in utilization of CDK4/6 inhibitors in the clinic, there is a need for better understanding of the metabolic consequences of CDK4/6 inhibition. In the current study, we report that activation of RB via treatment with the CDK4/6 inhibitor palbociclib in A549 lung adenocarcinoma cells results in a metabolic shift wherein palbociclib alters aspects of glucose and glutamine utilization. Specifically, palbociclib decreases glucose metabolism through the PPP via inhibition of G6PD activity (Fig. 3a–c), while increasing glutaminolysis to maintain basal mitochondrial function (Fig. 6c, f). Moreover, both changes observed were rescued upon knockdown of RB, suggesting the metabolic consequences of CDK4/6 inhibition in A549 cells are RB-dependent.

Recently, it has been demonstrated that CDKs can directly regulate metabolism via phosphorylation of metabolic enzymes. Specifically, the cyclin D3/CDK6 kinase complex can phosphorylate pyruvate kinase M2 and 6-phosphofructokinase [16]. The authors report this results in shunting of glycolytic intermediates into the PPP and the serine synthesis pathways, and treatment with palbociclib reduces this flux, resulting in NADPH and glutathione depletion and inducing apoptosis. We also observed a decrease in glucose carbon incorporation into nucleotides (Fig. 3a). This was in part due to decreased activity of the rate limiting enzyme in the PPP, G6PD (Fig. 3c). As A549 cells express both CDK4 and CDK6, it remains unclear whether the observed decrease in G6PD activity is due to the inhibitory effects of the cyclin D3/CDK6 complex.

As previously described, palbociclib increases mitochondrial activity in vitro [14, 15]. Specifically, CDK4/6 inhibition increases glutamine utilization and sensitivity to glutaminase inhibition in breast and colorectal cancer cells [15]. The authors reported that the increase in glutaminolysis was a result of enhanced MYC signaling in response to CDK4/6 inhibition. We did observe a modest increase in basal oxygen consumption upon palbociclib treatment (Fig. 4b), which is driven, in part, by enhanced glutaminolysis (Fig. 6c, f). This sensitized A549 cells to treatment with CB-839 (Fig. 6h). MYC exerts its effect on glutaminolysis in part via upregulation of glutaminase [39, 40]. As we did not observe a significant increase in *GLS* expression (Fig. 6g), our data suggest the enhanced glutaminolysis in this context may be independent of MYC signaling.

While other studies have reported enhanced glycolysis upon CDK4/6 inhibition or deletion, or direct activation of RB itself [14, 15, 41], our [U-^13^C]-glucose and ECAR studies revealed no significant changes in glycolytic function upon palbociclib treatment (Figs. 1, 2). One possible explanation for this discrepancy is the more acute treatment duration for our studies (48-h compared to 5–7 days). To address this, cells were treated with palbociclib for 5-days and, similar to 48-h exposure, we did not observe any differences in glycolytic parameters within the glycolysis stress test (Additional file 1: Figure S3). Prolonged CDK4/6 inhibition (96 h to 2 weeks) has been reported to induce both senescence and autophagy in vitro [14, 15, 42, 43]. Many of the reported metabolic consequences of longer-term palbociclib treatment are also metabolic hallmarks of these cellular processes. Senescent cells exhibit elevated glycolysis and mitochondrial metabolism, resulting in increased mitochondrial mass and production of reactive oxygen species (ROS) [44, 45]. This triggers autophagy and protein degradation to mitigate the cellular stress induced by senescence [46]. Recently, palbociclib-mediated induction of these cellular responses has been found to be dependent on both RB expression and lack of low molecular weight cyclin E (LMWE) [43]. As A549 cells express LMWE, this cell model is presumed to be resistant to senescence or autophagy upon palbociclib treatment [43]. This may explain the lack of glycolytic changes in A549 cells under prolonged exposure compared to other cell types [14, 15] and may need to be considered when assessing metabolic responses to palbociclib across different cancer models. Our studies are unique in that we have identified a metabolic response in A549 cells under shorter-term CDK4/6 inhibition.

Given the recent use of palbociclib in clinical trials for NSCLC patients, understanding the functional consequences of CDK4/6 inhibition on cancer cell metabolism are important for identifying potential combination therapies to improve patient outcome. Our studies in A549 cells demonstrate that palbociclib treatment increases glutamine dependency and sensitizes cells to the glutaminase inhibitor, CB-839. While the precise mechanism by which palbociclib mediates increased glutaminolysis remains unclear, RB expression is required for palbociclib to exert its function in this context. A noted limitation to this work is that the analysis on the metabolic response to palbociclib is solely performed within the A549 cell line. Further work will not only be necessary to define the cellular mechanisms for these palbociclib effects but also required in additional models to more firmly establish these metabolic changes in response to drug treatment across NSCLC in general. Our data highlight the metabolic changes upon palbociclib treatment in A549 cells and may support the of targeting CDK4/6 inhibition in combination with glutaminase inhibitors in NSCLC patients with RB-proficient tumors.

## Conclusions

Herein, we report that palbociclib treatments results in a metabolic shift in A549 cells. Specifically, we found that palbociclib decreases glucose metabolism through the PPP via inhibition of G6PD activity, while increasing dependency on glutaminolysis to maintain basal mitochondrial function While the precise mechanism by which palbociclib mediates its metabolic functions remain unclear, its effect on glucose and glutamine utilization in A549 cells are RB-dependent. Together, our data define the metabolic impact of CDK4/6 inhibition in a NSCLC cell type and highlight unique vulnerabilities that may be targeted using combination therapy with palbociclib treatment.

## Supplementary information

**Additional file 1: Figure S1.** Palbociclib decreases RB phosphorylation and proliferation of A549 lung cancer cells. **Figure S2.** Confirmation of siRNA-mediated knock-down of RB in A549 cells. **Figure S3.** Longer exposure to palbociclib does not alter glycolytic function in A549 cells.

## Data Availability

Not applicable.

## Abbreviations

NSCLC: Non-small cell lung cancer
RB: Retinoblastoma protein
CDK4/6: Cyclin-dependent kinase 4/6
PPP: Pentose phosphate pathway
ECAR: Extracellular acidification rate
G6PD: Glucose 6-phosphate dehydrogenase
PRPS2: Phosphoribosyl pyrophosphate synthetase 2
RRM1/2: Ribonucleotide reductase 1/2
OCR: Oxygen consumption rate
PDH: Pyruvate dehydrogenase
PC: Pyruvate carboxylase
GLS: Glutaminase
ROS: Reactive oxygen species
