# Supplementary material for: Palbociclib treatment alters nucleotide biosynthesis and glutamine dependency in A549 cells
Source: Cancer Cell Int. 2020 Jul 1;20:280. doi: 10.1186/s12935-020-01357-x (PMC7329430; doi:10.1186/s12935-020-01357-x)
Supplement: Supplementary file 1 — Additional file 1: Figure S1. Palbociclib decreases RB phosphorylation and proliferation of A549 lung cancer cells. Figure S2. Confirmation of siRNA-mediated knock-down of RB in A549 cells. Figure S3. Longer exposure to palbociclib does not alter glycolytic function in A549 cells. [file 12935_2020_1357_MOESM1_ESM.pdf]

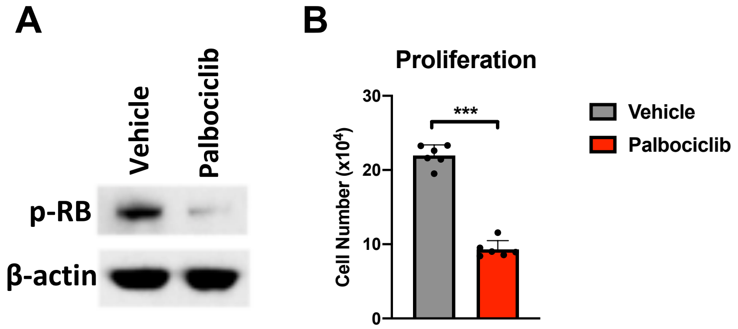

Additional File 1: Figure S1. Palbociclib decreases RB phosphorylation and proliferation of A549 lung cancer cells. (**A**) Western blot analysis of phosho-RB (p-RB) levels and (**B**) cell proliferation in A549 cells following 48-hour 1μM palbociclib treatment. β-actin was used as a loading control for immunoblot. Values represent mean ± SD, analyzed by unpaired t-test (n=5, independent experiments). Statistical significance between each group are as follows: ***$p<0.001$.

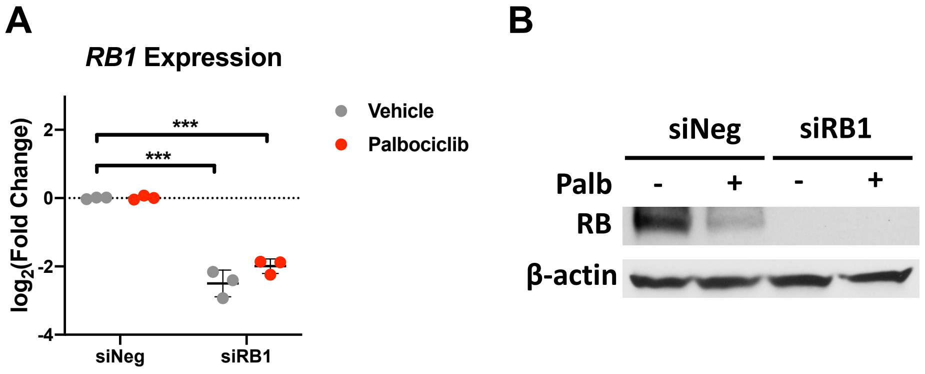

Additional File 1: Figure S2. Confirmation of siRNA-mediated knock-down of RB in A549 cells. (A) PCR and **(B)** immunoblot analysis of RB expression in A549 cells. Cells exposed to control (siNeg) or RB1-specific (siRB1) siRNA were treated with vehicle or 1μM palbociclib. β-actin was used as an internal or loading control for PCR and immunoblot analysis, respectively. Values represent mean ± SD, analyzed two-way ANOVA with Sidak's post-hoc multiple comparisons. Statistical significances between each group are as follows: ***p<0.001.

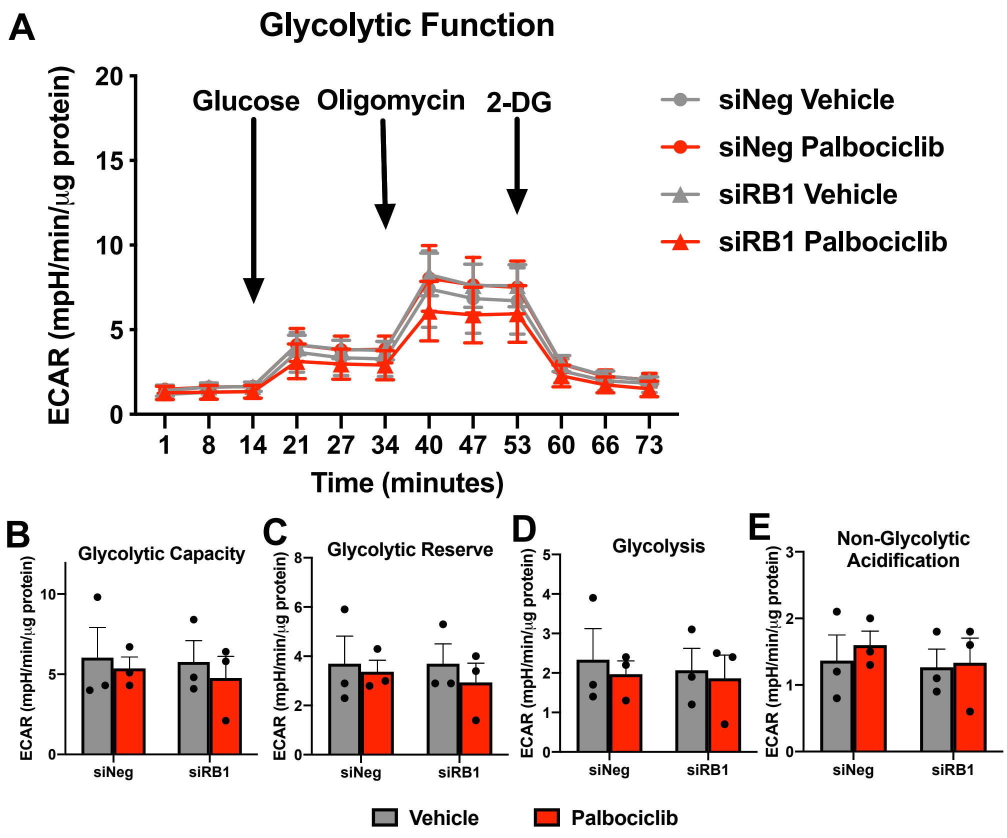

Additional File 1: Figure S3. Longer exposure to palbociclib does not alter glycolytic function in A549 cells. (**A**) Glycolysis stress test measured in A549 cells following knockdown of RB and 120-hour treatment with 1µM palbociclib using a Seahorse XFe96 Analyzer. (**B-E**) ECAR assessment of (**B**) glycolytic capacity, (**C**) glycolytic reserve, (**D**) glycolysis, and (**E**) non-glycolytic acidification. Values represent mean ± SD, analyzed by two-way ANOVA with Sidak's post-hoc multiple comparisons (n=3, independent experiments).
